# *Saireito* Improves Lymphatic Function and Prevents UVB-Induced Acute Inflammation and Photodamage in HR-1 Hairless Mice

**DOI:** 10.1155/2021/3707058

**Published:** 2021-06-25

**Authors:** Manami Oyama, Kenta Murata, Misaki Ogata, Nina Fujita, Ryuji Takahashi

**Affiliations:** Kampo Research Laboratories, Kracie Pharma, Ltd., 3-1 Kanebo-machi, Takaoka-shi, Toyama 933-0856, Japan

## Abstract

A single high-dose ultraviolet B (UVB) exposure on the skin induces acute inflammatory responses, such as an increase in proinflammatory cytokines (e.g., IL-6 and IL-1*β*), hyperpermeability and dilation of blood and lymphatic vessels, and infiltration of inflammatory cells. These responses result in different cutaneous disorders characterized by erythema, epidermal hyperplasia, edema formation, and extracellular matrix degradation. *Saireito* extract (SRT), a traditional Chinese medicine, has been used to treat various inflammatory diseases in Japan, and SRT and its major active components (e.g., saikosaponins and baicalin) were reported to downregulate proinflammatory cytokines. Moreover, SRT has a protective effect against UV irradiation in vitro. Based on these findings, we aimed to investigate the effect of SRT on UVB-induced photodamage and structural change in the vasculature. We pretreated male HR-1 hairless mice with SRT (625 or 1250 mg/kg) for 3 weeks before a single UVB (250 mJ/cm^2^) irradiation. SRT treatment attenuated UVB-induced increases in erythema, transepidermal water loss, and edema formation at 72 h after irradiation. SRT treatment also suppressed UVB-induced inflammatory cell infiltration and collagen degradation. Furthermore, at 24 h after irradiation, SRT treatment inhibited UVB-induced upregulation of proinflammatory cytokines and reduction in lymphatic vessel density associated with upregulation of VEGF-C expression. These results suggest that SRT could attenuate UVB-induced photodamage. This protective effect of SRT involves suppression of upregulation of proinflammatory cytokines and improvement of lymphatic function in the early stage of inflammation.

## 1. Introduction

The skin constitutes the outermost layer of the human body and plays a defensive role against physical, biological, and environmental hazards, including ultraviolet (UV) radiation. Acute UVB (280–320 nm) exposure induces a variety of cutaneous disorders, such as erythema, epidermal hyperplasia, edema formation, and alterations in the quantity and structure of the extracellular matrix (ECM) [1–5].

Among UVB-induced alterations in the ECM, collagen degradation is considered as the most severe damage because collagen constitutes the majority of ECM and has a slow turnover cycle. Collagen contributes to the strength and resilience of the skin, and intact and uniform collagen distribution plays an important role in maintaining healthy skin structure. However, UVB irradiation induces upregulation of matrix metalloproteinase (MMP) production and subsequent collagen degradation, resulting in wrinkle formation [6–8]. Furthermore, collagen fragmentation has been suggested to inhibit collagen and hyaluronan synthesis in photodamaged skin [9–11]. These observations indicate that collagen degradation triggers physical and physiological damage in UVB-irradiated skin.

This UVB-induced photodamage results from acute inflammatory responses, such as an increase in proinflammatory cytokines (e.g., IL-6 and IL-1*β*) [4, 12, 13], and the blood and lymphatic vasculature play an important role in the progression of inflammation. UVB irradiation induces vascular hyperpermeability and blood vessel dilation via production of prostaglandin E2 (PGE2) [14], resulting in increased extravasated fluid including excess water, macromolecules, and inflammatory cells. Although lymphatic vessels play a crucial role in the drainage of extravasated fluid and maintenance of tissue fluid homeostasis [15], lymphatic vessels are reduced in photo-aged skin [16]. Moreover, UVB irradiation induces the hyperpermeability of cutaneous lymphatic vessels [17]. The intradermal injection of vascular endothelial growth factor C (VEGF-C), a key molecule in lymphangiogenesis, was reported to suppress edema formation and promote recovery from inflammation by increasing lymphatic vessel density in a UVB-induced inflammation model [18]. These findings suggest that the improvement of vasculature impairment is a potential strategy for attenuating UVB-induced skin inflammation.

*Saireito* extract (SRT), a traditional Chinese medicine extracted from a mixture of 12 crude drugs, has been traditionally prescribed in Japan for patients with watery diarrhea, acute gastroenteritis, heatstroke, and edema with symptoms of nausea, hypophagia, thirst, and oliguria. Saikosaponins and baicalin are indicator ingredients and the major active compounds of SRT. These compounds were suggested to downregulate proinflammatory cytokines in different inflammatory diseases [19–21], and topical application of baicalin was reported to ameliorate UVB-induced photodamage [22]. Furthermore, SRT was suggested to improve lymphedema after radiotherapy in a clinical study [23] and was found to have a protective effect against UV irradiation in the human immortalized skin keratinocyte [24]. However, it has not been investigated whether and how oral administration of SRT has a protective effect on UVB-induced damage in vivo. HR-1 hairless mice have been used to evaluate the protective effect against UVB-induced photodamage and structural change in vasculature [17, 25]. Hence, in this study, we aimed to investigate the effect of SRT on UVB-induced damage and vasculature change in HR-1 hairless mice.

## 2. Materials and Methods

### 2.1. Plant Materials and Extract Preparation

SRT is an extract of 12 crude drugs (Table 1) and is standardized according to the quality and quantity of ingredients by the Japanese Pharmacopoeia [26]. It was supplied by Kracie Pharma, Ltd. (Tokyo, Japan), as a formulation (KB-114). The dosage of KB-114 for an adult human is 8100 mg/day, containing 7000 mg of SRT extracted from the amounts of crude drugs shown in Table 1. KB-114 (Lot no. 02EH) was suspended in distilled water immediately before use.

### 2.2. High-Performance Liquid Chromatography Analysis of SRT

SRT extract was mixed and shaken with 50% MeOH and centrifuged, and the supernatant was analyzed using high-performance liquid chromatography (HPLC). The three-dimensional- (3D-) HPLC profile of SRT was obtained using the Shimadzu LC-30AD liquid-chromatography system (Shimadzu Corporation, Kyoto, Japan) equipped with the SPD-M30A detector (Shimadzu Corporation) with a scanning range of 200–700 nm and a reversed-phase column (YMC-Triart C18, 3.0 mm i.d. ×50 mm, 12 nm; YMC Co., Ltd., Kyoto, Japan; column temperature, 20°C). The column was filled with solvent A (0.1% formic acid in acetonitrile) and solvent B (0.1% formic solution), and the ratio of solvent A was increased from 15% to 50% over 60 min with a flow rate of 0.2 mL/min.

### 2.3. Experimental Animals and Sample Treatment

Five-week-old male HOS:HR-1 hairless mice were purchased from Japan SLC, Inc. (Shizuoka, Japan) and were housed in sterilized polypropylene cages (4–5 mice/cage) with paper bedding (Japan SLC, Inc.) at 24 ± 2°C under a 12 h light-dark cycle (lights on from 8 : 00 to 20 : 00) and allowed food and water ad libitum. All efforts were made to minimize both animal suffering and the number of animals used. This study was conducted in accordance with the principles of the Basel Declaration and recommendations of guidelines for proper conduct of animal experiments from the Experimental Animal Care Committee of Kracie Pharma, Ltd. The experimental protocol was reviewed and approved by the Experimental Animal Care Committee of Kracie Pharma, Ltd. (approval number, 180040). During housing, no adverse events were observed. After 1 week of acclimatization, the mice weighed (mean ± SD) 21.4 ± 1.3 g and were divided randomly into four groups using the standard randomization function in Microsoft Excel: control (*n* = 5), vehicle (*n* = 5 at each time point), low-dose SRT (625 mg/kg; *n* = 4), and high-dose SRT groups (1250 mg/kg; *n* = 5 at each time point). The total number of mice was 39. The sample size and protocols were considered and determined based on a preliminary experiment. The experimental unit is the individual animal. The dose was determined with reference to the formula as follows: dB = dA × KB/KA. dB is the daily dose, in milligram, per kilogram of mouse body weight. dA is the daily dose, in milligram, per kilogram of adult human body weight. The final term is a constant, with KB = 1.0 and KA = 0.11. For the calculation, the following values were used: normal KB-114 dosage for an adult human, 8100 mg/day, and adult human weight, 60 kg. Converted into dosage for mice, this yields 1227 mg/kg mouse body weight/day. SRT suspension was administered orally 5 days a week for 3 weeks, and the mice in the control and vehicle groups were given distilled water for the administration period. Each mouse was given a random number, and only one investigator who administered the treatment was aware of the treatment group. All analyses were based on the random number and performed by another investigator.

### 2.4. UVB Irradiation and Measurement of Erythema Index and Transepidermal Water Loss

One hour after the last administration, mice were anesthetized with isoflurane and irradiated using a handheld UV lamp (UVB-57; Analytik Jena, Jena, Germany), which has a peak irradiance at 302 nm. The UVB dose was measured using the digital UV measuring instrument UV-340A (Lutron Electronic Enterprise Co., Ltd., Taipei, Taiwan). UVB radiation of 250 mJ/cm^2^ and a 72 h observation period were adopted because of significant increases in erythema and transepidermal water loss in the preliminary experiment. Mice were placed 12 cm underneath the UV lamp and exposed to 250 mJ/cm^2^ UVB radiation in the dorsal skin. The humane endpoints established for the mice were rapid weight loss of more than 20%, self-injury due to severe pain, and abnormality of irradiated sites. Before and every 24 h after UVB irradiation, the erythema index (EI) and transepidermal water loss (TEWL) were measured under isoflurane anesthesia using Mexameter MX18 and Tewameter TM300 (Courage and Khazaka Eletronic, Cologne, Germany), respectively. The EI was calculated automatically based on absorbance of hemoglobin. The probe of MX18 emits and receives specific wavelengths (green, 568 nm; red, 660 nm) corresponding to the spectral absorption peak of hemoglobin and to avoid other color influences. The order of measurements was randomized at each time point. After the measurements, mice were sacrificed under isoflurane anesthesia, and their dorsal skin was collected. Half of the skin sample was immersed in Bouin's solution for histochemical analysis, and the other half was stored at −80°C to measure protein and mRNA expression level. ΔEI and ΔTEWL were calculated by subtracting the value after UVB irradiation with the value before irradiation.

### 2.5. Histological Analysis

The dorsal skin tissue fixed in Bouin's solution overnight at 4°C was dehydrated in ethanol (50–100%), cleared in xylene, embedded in paraffin wax, and sectioned at 5 *μ*m thickness. Subsequently, the deparaffinized sections were stained with hematoxylin and eosin (HE) and Masson's trichrome stain to evaluate skin thickness and collagen fibers, respectively. Each section was examined under the Axio Observer.Z1/7 light microscope (ZEISS, Jena, Germany) at 10x magnification. Epidermal thickness was calculated by dividing the area of the epidermis by the length of the basal layer, whereas dermal thickness was obtained by dividing the area of the dermis by the length of the muscle. Collagen density was calculated using Fiji software [27].

### 2.6. Immunohistochemical Analysis

For the staining of Iba-1 and Ly-6G/6C, endogenous peroxidases were inhibited by incubating the section for 30 min at 24 ± 2°C with freshly prepared 0.3% hydrogen peroxide in methanol. The sections were subsequently treated with citrate buffer (pH 6.0) overnight at 60°C for antigen retrieval. Nonspecific staining was blocked with 5% goat serum for 1 h at 24 ± 2°C, and the sections were then incubated with rabbit polyclonal anti-Iba-1 antibody (1 : 2000; Abcam, Beverly, MA, USA) or rat monoclonal anti-Ly-6G/6C antibody (1 : 400; Abcam) overnight at 4°C. The sections were washed with phosphate-buffered saline (PBS), incubated at 24 ± 2°C for 1 h with HRP-conjugated anti-rabbit secondary antibody or anti-rat secondary antibody (MDB Co., Ltd., Tokyo, Japan), developed by incubating with diaminobenzidine peroxidase substrate solution (Sigma-Aldrich, St. Louis, MO, USA), and counterstained with hematoxylin solution.

For podoplanin immunostaining, sections were treated with citrate buffer (pH 6.0) overnight at 60°C, blocked with mouse IgG blocking reagent (Vector Laboratories, Inc., Burlingame, CA, USA) for 1 h at 24 ± 2°C to eliminate nonspecific staining, and then incubated with mouse monoclonal anti-podoplanin antibody (1 : 200; GeneTex, Beverly, MA, USA) in Can Get Signal® immunostain Immunoreaction Enhancer Solution A (TOYOBO Co., Ltd., Osaka, Japan) overnight at 4°C. After washing with PBS, the sections were incubated with anti-mouse secondary antibody labeled with Alexa fluor-594 (Invitrogen, Waltham, MA, USA) for 1 h at 24 ± 2°C and counterstained with DAPI.

For Meca-32 immunostaining, sections were treated with Tris-EDTA buffer (pH 9.0) overnight at 60°C, blocked with 5% goat serum to eliminate nonspecific staining, and then incubated with rat monoclonal anti-Meca-32 antibody (BD Pharmingen, Franklin Lakes, NJ, USA) overnight at 4°C. After washing with PBS, the sections were incubated with anti-rat secondary antibody conjugated with Alexa fluor-488 for 1 h at 24 ± 2°C and counterstained with DAPI.

Images were taken under the Axio Observer.Z1/7 light or fluorescence microscope at 10x magnification. Tissues were stained at the same time for each antibody, and those that were severely damaged during the staining process were excluded from the analysis. The densities of cells positive for Iba-1 and Ly-6G/6C and lymphatic and blood vessels were manually obtained using the Fiji software. The sizes of the lymphatic or blood vessels were measured by dividing the area of lymphatic or blood vessels by the counted number.

### 2.7. PGE2 Measurement

Skin PGE2 level was measured by performing ELISA with the Enzyme Immunoassay Kit Correlate PGE2 (Assay Designs Inc., Ann Arbor, MI, USA) and following the product manual. In brief, frozen skin tissues were minced and homogenized with 10 *μ*g/mL indomethacin. Subsequently, the lysate was centrifuged at 13,000x g at 4°C for 20 min, and supernatants were collected. Diluted standards and samples were applied to the ELISA kit.

### 2.8. Quantitative Real-Time PCR

Real-time PCR was performed to measure the expression of IL-6 and IL-1*β* mRNA. Frozen skin tissues including the epidermis and dermis were minced on dry ice and homogenized in lysis buffer containing mercaptoethanol. Total RNA was isolated from the lysate using GenElute™ Mammalian Total RNA Miniprep Kit (Sigma-Aldrich), and mRNA was reverse-transcribed into cDNA by using Rever Tra Ace qPCR RT Master Mix with gDNA Remover (TOYOBO Co., Ltd.). Quantitative real-time PCR was performed using TB Green^®^*Premix EX Taq*^TM^ II (Tli RNaseH Plus) (Takara-bio, Shiga, Japan) with the following amplification protocol: 40 cycles of 95°C for 5 s and 60°C for 30 s; a single fluorescence measurement was used. Melting-curve analysis, for which the temperature was increased from 60 to 95°C at a heating rate of 0.1°C/s and continuous fluorescence measurement was used, revealed a single, narrow peak of the suspected fusion temperature. The primer sequences used are shown in Table 2. The expression of target genes was normalized to that of *β*-actin, and the fold changes of mRNA expression were calculated by dividing the test mRNA expression level by the control expression level.

### 2.9. Western Blot Analysis

Frozen skin tissues including the epidermis and dermis were minced and homogenized in a radioimmunoprecipitation assay lysis buffer (WAKO, Osaka, Japan) containing protease inhibitor cocktail (Nakalai Tesque, Kyoto, Japan) and phosphatase inhibitor cocktail (Nakalai Tesque). The lysate was centrifuged at 15,000x g for 20 min at 4°C, and supernatants were used to determine the protein concentration using the BCA protein assay kit (Thermo Fisher Scientific, Waltham, MA, USA). Tissue extracts containing 10 *μ*g of proteins were subjected to electrophoresis on 10–20% SDS-PAGE gel and transferred to a polyvinylidene difluoride (PVDF) membrane (Immobilon-P; Millipore, Burlington, MA, USA) using transblot semidry apparatus. PVDF membranes were blocked with 5% nonfat milk for 1 h and then incubated with rabbit polyclonal anti-VEGF-C antibodies (1 : 1000; GeneTex) or mouse monoclonal anti-*β*-actin antibody (1 : 1000; Cell Signaling Technology, Danvers, MA, USA) overnight at 4°C. The PVDF membranes were washed with tris-buffered saline and Tween 20 (TBS-T) and incubated with HRP-conjugated goat anti-rabbit IgG or goat anti-mouse IgG (1 : 5000; Cell Signaling Technology) for 1 h at 24 ± 2°C. Subsequently, the membranes were washed with TBS-T, and the developed bands were detected using a chemiluminescence kit. Immunoreactive bands were visualized using the Amersham Imager 680 (GH Healthcare, Chicago, IL, USA). The relative intensity of protein bands was normalized to *β*-actin expression. The fold changes of protein expression were calculated by dividing the test protein intensity by the control protein intensity.

### 2.10. Statistical Analysis

All data are expressed as mean ± standard error of the mean (SEM). Statistical comparisons were performed using one-way analysis of variance followed by Student's *t*-test or Dunnett's test (Statcel, OMS Ltd., Saitama, Japan). For all tests, differences with *p* < 0.05 were considered statistically significant.

## 3. Results

### 3.1. HPLC Analysis of SRT

**Figure 1:**
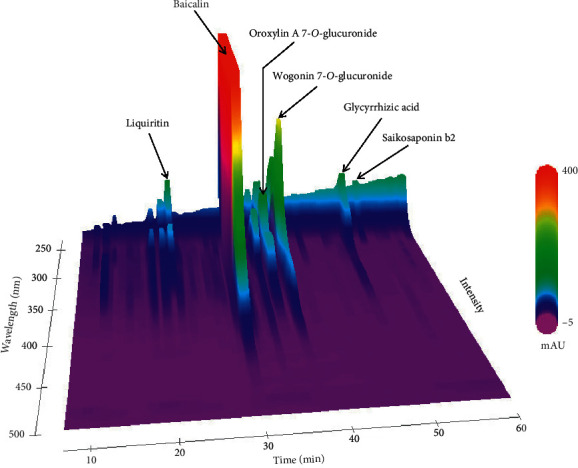
3D-HPLC profile of SRT. Chemical markers (liquiritin, baicalin, oroxylin A 7-O-glucuronide, wogonin 7-O-glucuronide, glycyrrhizic acid, and saikosaponin b2) in HPLC profiles were identified based on comparison with the retention times and UV spectra (200–700 nm) of their reference standards. HPLC: high-performance liquid chromatography; SRT: *Saireito* extract; UV: ultraviolet; AU: absorbance unit.

Figure 1 shows a 3D-HPLC profile of SRT together with a chemical analysis. Chemical markers, such as liquiritin, baicalin, oroxylin A 7-O-glucuronide, wogonin 7-O-glucuronide, glycyrrhizic acid, and saikosaponin b2, were used for quality control.

### 3.2. SRT Treatment Suppressed UVB-Induced Increase in EI and TEWL

**Figure 2:**
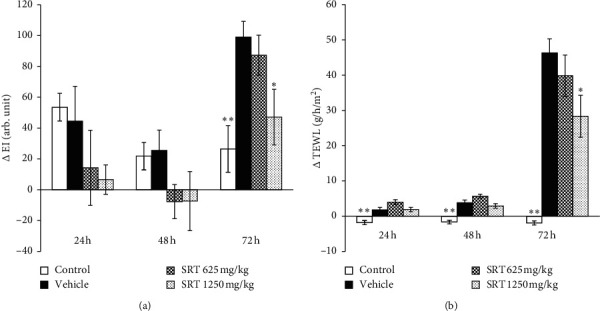
Effect of SRT on UVB-induced skin damage. Mice were pretreated with SRT (625 or 1250 mg/kg) 5 days a week for 3 weeks before UVB irradiation, as described in Section 2. SRT suppressed UVB-induced increase in EI (a) and TEWL (b) at 72 h after irradiation. Data were expressed as mean ± SEM (*n* = 4-5). ^*∗*^*p* < 0.05 and *∗∗p* < 0.01 vs. vehicle, Dunnett's test. SRT: *Saireito* extract; UVB: ultraviolet B; EI: erythema index; TEWL: transepidermal water loss; arb. unit: arbitrary unit.

We initially evaluated whether SRT has a preventive effect on UVB-induced skin damage in hairless mice. We treated HR-1 hairless mice with distilled water or SRT suspension (625 or 1250 mg/kg) for 3 weeks before UVB irradiation (250 mJ/cm^2^) and measured EI and TEWL before and every 24 h after UVB irradiation for 3 days. Consequently, a single UVB irradiation significantly increased EI at 72 h after irradiation, and pretreatment with SRT (1250 mg/kg) significantly inhibited the increase in EI as compared with the vehicle group (Figure 2(a)). In addition, TEWL was significantly increased from 24 h after irradiation, and a substantial increase was detected at 72 h compared with the control group. Pretreatment with SRT (1250 mg/kg) significantly inhibited the increase in TEWL compared with the vehicle group at 72 h, but not at 24 and 48 h after irradiation (Figure 2(b)).

### 3.3. SRT Treatment Attenuated UVB-Induced ECM Degradation

**Figure 3:**
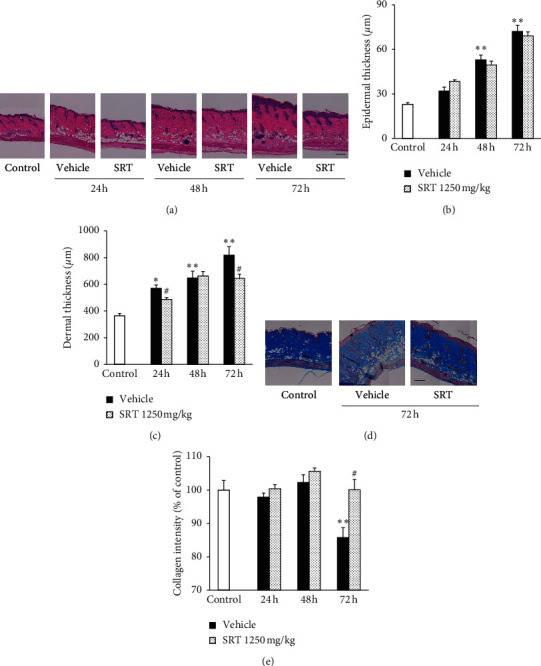
Effect of SRT on UVB-induced skin thickness and collagen degradation. (a) Skin sections were collected every 24 h after irradiation, and HE staining was performed. (b) SRT had no effect on UVB-induced epidermal hyperplasia. (c) SRT significantly inhibited UVB-induced edema formation at 24 h and 72 h after irradiation. (d) Skin sections were collected at 72 h after irradiation, and Masson's trichrome staining was performed. (e) SRT significantly attenuated UVB-induced decrease of collagen fibers at 72 h after irradiation. Data were expressed as mean ± SEM (*n* = 5). Scale bar = 200 *μ*m. ^*∗*^*p* < 0.05 and ^*∗∗*^*p* < 0.01 vs. control, Dunnett's test. ^#^*p* < 0.05 vs. vehicle, Student's *t*-test. SRT: *Saireito* extract; UVB: ultraviolet B.

UVB-irradiated skin is histologically characterized by collagen degradation, epidermal hyperplasia, and edema formation [2, 27, 28]. To evaluate the effects of SRT on UVB-induced skin thickening, we stained the UVB-irradiated skin with HE (Figure 3(a)). The thickness of the epidermis and dermis was serially increased after UVB irradiation. SRT treatment (1250 mg/kg) significantly decreased the UVB-induced thickening in the dermis at 24 h and 72 h after irradiation, but not in the epidermis (Figures 3(b)and 3(c)). Furthermore, we used Masson's trichrome staining to evaluate the effects of SRT on UVB-induced collagen degradation (Figure 3(d)). A single UVB irradiation significantly reduced the density of collagen fibers at 72 h after irradiation, and SRT treatment (1250 mg/kg) significantly suppressed the reduction of collagen fibers (Figure 3(e)).

### 3.4. SRT Treatment Suppressed Infiltration of Inflammatory Cells

**Figure 4:**
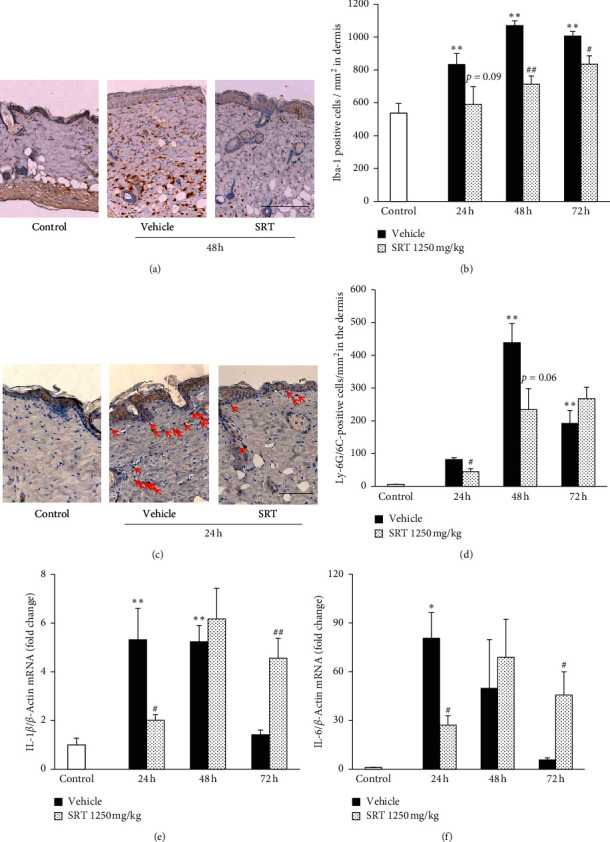
Effect of SRT on UVB-induced inflammatory cell infiltration and proinflammatory cytokine production. (a) Skin sections were collected at 48 h after irradiation, and immunohistochemical staining was performed for Iba-1. Scale bar = 200 *μ*m. (b) SRT significantly inhibited UVB-induced infiltration of macrophages at 48 h and 72 h. (c) Skin sections were collected at 24 h after irradiation, and immunohistochemical staining was performed for Ly-6G/6C. Arrows indicate Ly-6G/6C-positive cells in the dermis. Scale bar = 100 *μ*m. (d) SRT significantly inhibited neutrophil infiltration at 24 h after irradiation. (e) and (f) SRT significantly inhibited UVB-induced increase in IL-1*β* and IL-6 at 24 h after irradiation. On the contrary, SRT increased the expression level of IL-1*β* and IL-6 at 72 h compared with the vehicle group. Data were expressed as mean ± SEM (*n* = 4-5). ^*∗*^*p* < 0.05 and ^*∗∗*^*p* < 0.01 vs. control, Dunnett's test. ^#^*p* < 0.05 and ^##^*p* < 0.01 vs. vehicle, Student's *t*-test. SRT: *Saireito* extract; UVB: ultraviolet B.

UVB exposure induces infiltration of macrophages and neutrophils, which are involved in ECM degradation [29–31]. Afterward, we assessed the effect of SRT on inflammatory cell infiltration in the UVB-irradiated skin. To evaluate the effect on macrophage infiltration into the dermis, we performed immunostaining for the macrophage marker Iba-1 (Figure 4(a)). UVB increased the number of Iba-1-positive cells from 24 h after irradiation, and SRT treatment (1250 mg/kg) suppressed this increase at 48 h and 72 h after irradiation (Figure 4(b)). In addition to macrophage infiltration, UVB irradiation increased neutrophil infiltration (Ly-6G/6C-positive cells) at 48 h and 72 h after irradiation. SRT treatment reduced neutrophil infiltration at 24 h and 48 h compared with the vehicle group; however, the difference was not statistically significant at 48 h (*p* = 0.06) (Figure 4(d)).

### 3.5. SRT Treatment Inhibited Production of Proinflammatory Cytokines

Next, we investigated the mRNA expression level of proinflammatory cytokines (IL-1*β* and IL-6) to reveal the mechanism by which SRT treatment suppressed skin inflammation. UVB irradiation significantly enhanced the expression level of IL-6 mRNA at 24 h and IL-1*β* mRNA at 24 h and 48 h after irradiation compared with the control group. SRT treatment (1250 mg/kg) significantly attenuated the expression level of IL-1*β* and IL-6 mRNA at 24 h after irradiation. In contrast, IL-1*β* and IL-6 mRNA expression level significantly increased in the SRT group compared with the vehicle group at 72 h after irradiation (Figures 4(e)and 4(f)).

### 3.6. SRT Treatment Had No Effect on UVB-Induced Vasodilatation and PGE2 Production

**Figure 5:**
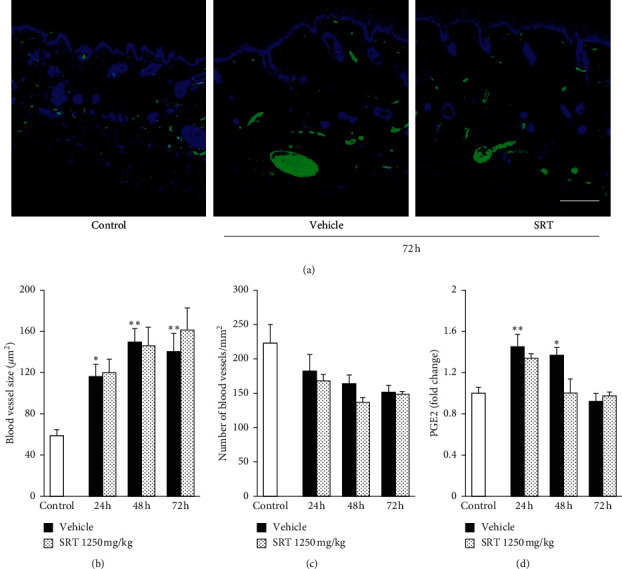
Effect of SRT on UVB-induced structural change in blood vessels and PGE2 contents. (a) Skin sections were collected at 72 h after irradiation, and immunohistochemical staining was performed for the blood vessel marker Meca-32. Scale bar = 200 *μ*m. (b) UVB irradiation increased the blood vessel size from 24 h to 72 h after irradiation, and SRT had no effect on dilation. (c) UVB irradiation and SRT treatment had no effect on blood vessel density. (d) PGE2 expression significantly increased at 24 h and 48 h after irradiation, and SRT had no effect on the UVB-induced upregulation of PGE2. Data were expressed as mean ± SEM (*n* = 4-5). ^*∗*^*p* < 0.05 and ^*∗∗*^*p* < 0.01 vs. control, Dunnett's test. SRT: *Saireito* extract; UVB: ultraviolet B; PGE2: prostaglandin E2.

The vascular system comprising the blood vessels and lymphatic vessels plays an important role in the regulation of UVB-induced inflammation. We initially focused on the structural change in the blood vessels. UVB irradiation was reported to induce vasodilatation and vascular hyperpermeability via PGE2 production, resulting in erythema induction and increased inflammatory cell infiltration [14, 32]. To evaluate the effect of SRT on the blood vessels, we performed immunostaining for Meca-32 as a blood vessel marker (Figure 5(a)). As a result, UVB irradiation induced a significant increase in blood vessel size from 24 h to 72 h after irradiation, but not in blood vessel number (Figures 5(b)and 5(c)). PGE2 production was significantly increased at 24 h and 48 h after UVB irradiation compared with the control group (Figure 5(d)). Pretreatment with SRT (1250 mg/kg) had no effect on UVB-induced structural change in blood vessels and PGE2 content.

### 3.7. SRT Treatment Suppressed UVB-Induced Reduction in the Number of Lymphatic Vessels

**Figure 6:**
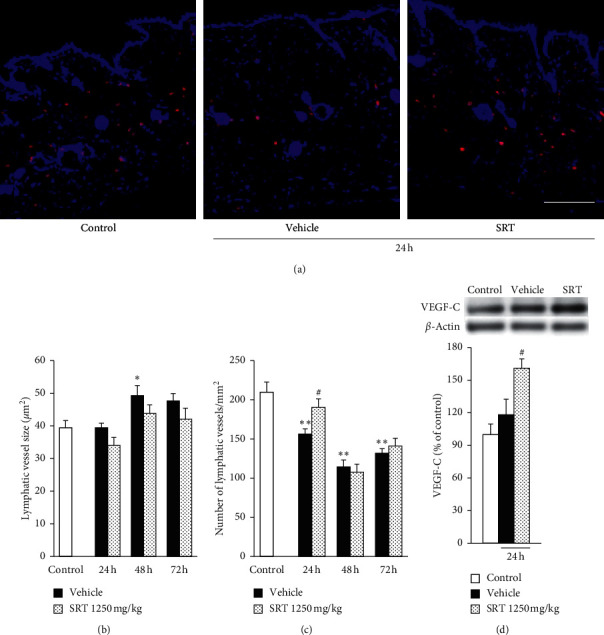
Effect of SRT on UVB-induced structure change in lymphatic vessels and VEGF-C expression level. (a) Skin sections were collected at 24 h after irradiation, and immunohistochemical staining was performed for the lymphatic vessel marker podoplanin. Scale bar = 200 *μ*m. (b) UVB irradiation increased lymphatic vessel size at 48 h after irradiation, and SRT had no effect on the dilation. (c) SRT suppressed UVB-induced decrease in lymphatic vessel density at 24 h after irradiation. (d) VEGF-C expression at 24 h after UVB irradiation was measured using western blot analysis. SRT upregulated the VEGF-C expression compared with the vehicle group at 24 h after irradiation. Data were expressed as mean ± SEM (*n* = 5). ^*∗*^*p* < 0.05 and ^*∗∗*^*p* < 0.01 vs. control, Dunnett's test. ^#^*p* < 0.05 vs. vehicle, Student's *t*-test. SRT: *Saireito* extract; UVB: ultraviolet B; VEGF-C: vascular endothelial growth factor C.

We investigated whether SRT has an effect on lymphatic vessel structure. Lymphatic vessels drain excess water and inflammatory cells; thus, they play an important role in controlling edema formation and inflammation in UVB-irradiated skin [33]. To investigate the effect of SRT on the lymphatic structure, we performed immunostaining for podoplanin as a lymphatic vessel marker (Figure 6(a)). UVB irradiation induced a significant increase in lymphatic vessel size at 48 h and reduced lymphatic vessel density from 24 h to 72 h after irradiation. SRT treatment (1250 mg/kg) suppressed UVB-induced reduction of lymphatic vessel density at 24 h after irradiation; however, it did not suppress an increase in lymphatic vessel size (Figures 6(b)and 6(c)). Injection of VEGF-C was reported to promote lymphangiogenesis and recovery in the UVB-irradiated skin inflammation model [18]. To investigate the mechanism on how SRT suppressed UVB-induced reduction of lymphatic vessel density, we measured the expression level of VEGF-C in the skin at 24 h after irradiation. As a result, the expression level of VEGF-C significantly increased in the SRT group, but not in the vehicle group (Figure 6(d)).

## 4. Discussion

In this study, we demonstrated that SRT had a protective effect against UVB-induced erythema, edema formation, and collagen degradation. SRT also inhibited UVB-induced infiltration of inflammatory cells and cytokine production. Finally, SRT partially modified lymphangiogenesis.

In the UVB-induced inflammation model, cutaneous vasculature was reported to play an important role in edema formation. UVB irradiation facilitates vascular hyperpermeability and blood vessel dilation in the skin [17], resulting in extravasated fluid including excess water, macromolecules, and inflammatory cells. In this study, UVB irradiation induced blood vessel dilation, infiltration of neutrophils and macrophages, and edema formation from 24 h after irradiation (Figures 3(c), 4(a)–4(d), and 5(b)). At the wound site, neutrophils are reported to infiltrate the wound quickly and begin to wane by apoptosis in the early inflammation phase. Concomitantly with the influx of neutrophils, circulating monocytes enter the damaged area and differentiate into mature tissue macrophages and play an important role in both activation of inflammation and wound healing during inflammation and proliferation phase [34, 35]. Consistent with these reports, neutrophils significantly increased at 48 h after irradiation and decreased to less than half at 72 h after irradiation, whereas macrophages remained at high level from 24 h to 72 h after irradiation. At 24 h after irradiation, SRT inhibited edema and neutrophil infiltration and tended to suppress macrophage infiltration compared with the vehicle group (Figures 3(c)and 4(a)–4(d)). However, SRT had no effect on blood vessel structure. Given that lymphatic vessels play a central role in the clearance of extravasated fluid, they are recognized as an important factor in resolving inflammation. The density of lymphatic vessels significantly decreases in photodamaged human skin [16], and UVB irradiation induces hyperpermeability of lymphatic vessels in the mice model [17]. Among lymphangiogenic factors, VEGF-C is regarded as the key molecule that promotes proliferation and migration of lymphatic endothelial cells [36, 37]. In the acute inflammation model, VEGF-C overexpression accelerates the migration of inflammatory cells from the inflamed skin to the lymph node, resulting in less swelling and erythema [38]. Moreover, in the UVB-induced skin inflammation model, VEGF-C injection was reported to increase the density of lymphatic vessels and inhibit the infiltration of macrophages, resulting in early recovery from UVB-induced edema [18]. In this study, UVB irradiation significantly decreased lymphatic vessel density, and no difference was found in VEGF-C expression level in the vehicle group compared with the control group at 24 h after irradiation. SRT treatment suppressed this reduction of lymphatic vessel density accompanied with upregulation of VEGF-C expression (Figure 6). This result suggests that SRT improves drainage capacity of lymphatic vessels, resulting in suppression of edema at 24 h after irradiation. VEGF-C has been reported to bind to VEGF receptor 3 (VEGFR3) expressed on lymphatic endothelial cells (LECs) and, in addition, bind to VEGFR2 distributed on both LECs and blood vascular endothelial cells (BECs). However, the effect of VEGF-C on the blood vasculature is minimal in many models [39]. Correspondingly, in our study, VEGF-C upregulation by SRT affected only the lymphatic vessel density. It was reported that proteins which are required for the activation of VEGF-C are located primarily on the surface of LECs and only slightly expressed on the surface of BECs [40]. This specificity of localization could explain why VEGF-C preferentially activated lymphangiogenesis. Several inflammatory mediators have been found to induce VEGF-C transcription, and inflammation is commonly associated with lymphangiogenesis [41]. In UVB-irradiated model, however, it was reported that the expression of VEGF-C is not upregulated despite activation of inflammation [18], and how UVB irradiation suppressed the VEGF-C expression remains to be clarified. Further studies are necessary to reveal the mechanism by which SRT upregulated VEGF-C expression. Many studies have suggested that UVB irradiation upregulates proinflammatory cytokines, such as IL-1*β* and IL-6, and these cytokines are responsible for the onset of inflammation and the induction of neutrophil and macrophage infiltration [4, 29, 42, 43]. Oral administration of SRT was reported to downregulate IL-1*β* mRNA in mice intestinal mucositis model [44]. Saikosaponins are a well-known active component of SRT and were reported to suppress the upregulation of proinflammatory cytokines, including IL-1*β* and IL-6 in macrophages via inhibition of NF-*κ*B and mitogen-activated protein kinase [19, 20]. In this study, we showed that SRT inhibits the increase in IL-1*β* and IL-6 mRNA at 24 h after irradiation (Figures 4(e)) and 4(f)). In addition, IL-1*β* and IL-6 were reported to enhance lymphatic vessel permeability by affecting the junctional and contractile components of permeability and to downregulate the pumping activity of lymphatic vessels [45, 46]. These results suggest that SRT treatment may improve UVB-induced lymphatic dysfunction by inhibiting proinflammatory cytokine production. The lymphatic capillaries are connected to the surrounding ECM by anchoring filaments, which attach to collagen fibers [15]; hence, lymphatic vessels should dilate with edema formation, and single UVB irradiation was reported to induce significant dilation of lymphatic vessels from 24 h after irradiation in the ears of mice [18]. However, in our dorsal skin model, despite the significant hyperplasia of dermis from 24 h after irradiation, the dilation of lymphatic vessels was observed only at 48 h after irradiation, and the rate of dilation was less severe than that previously reported (Figure 6(b)). This result may be attributed to the difference in the irradiated site, and the reduction of lymphatic vessel density in UVB-irradiated skin suggests that UVB-induced fragmentation of collagen fibers might reduce the attachment of lymphatic endothelium to ECM, resulting in rarefication of lymphatic vessels.

Erythema is often observed after UVB irradiation and is measured to determine the degree of inflammation. UVB irradiation upregulates COX-2 expression which mediates synthesis of PGE2 from arachidonic acid [47]. PGE2 induces vasodilation and increase in blood flow by stimulation of the receptor, resulting in induction of erythema [14, 32]. We observed the gradual expansion of blood vessels associated with the increase in PGE2 content, and this expansion continued for at least 72 h after UVB irradiation (Figure 5). Despite continuous vasodilation from 24 h after irradiation, the increase in EI was detected only at 72 h after UVB irradiation (Figure 2(a)). Moreover, PGE2 contents were increased at 24 and 48 h after irradiation, but not at 72 h after irradiation when the EI was increased. Although SRT and saikosaponins were reported to suppress PGE2 production in LPS-stimulated macrophage [19, 48], no difference was found in the secretion of PGE2 between the SRT and vehicle groups after UVB irradiation. These results indicate that the increase in EI at 72 h after irradiation might be affected by factors other than blood vessel structure and PGE2. Although further work is necessary to elucidate the mechanism of UVB-induced erythema, a possible mechanism might be associated with reduction of collagen density observed at 72 h after irradiation (Figures 3(d)–3(e)). Collagen fibers, which constitute the bulk (70% dry weight) of the dermis, are stabilized by cross-links and constitute a uniform structure in the healthy skin. In contrast, collagen fragmentation, clumping of fragmented collagen, and irregular collagen distribution have been observed in photodamaged skin [49]. Furthermore, collapse of ECM might affect the water-holding capacity of the skin. Since TEWL is defined as the volume of water that passes from inside to outside of the body through the epidermal layer, it is considered to reflect the barrier function of the horny layer. In our study, however, SRT had no effect against UVB-induced hyperplasia in the epidermis (Figure 3(d)). The collagen fragments in the dermis not only contribute to the heterogeneous structure of the skin in fibroblasts after UVB irradiation but also inhibit the synthesis of hyaluronan [11], which is a major contributor to the water content in the skin. In the present study, we showed that SRT treatment prevented UVB-induced edema, erythema, TEWL impairment, and reduction of collagen density at 72 h after irradiation (Figures 2 and 3). In addition, no difference was found in lymphatic vessel structure between the vehicle and SRT groups at 72 h after irradiation (Figure 6(c)). These results suggest that SRT might improve the edema, erythema, and TEWL impairment by inhibiting ECM degradation at 72 h after irradiation and that this mechanism is different from that in 24 h after irradiation.

Within the ECM, the major structural proteins are collagen and elastin, and proteolytic enzymes, such as MMP and elastases, which are produced by epidermal keratinocytes and fibroblasts, mediate ECM remodeling. MMP production and subsequent collagen alteration are observed in photoaging and aged skin, and UVB irradiation was reported to alter the signal transduction pathways that promote MMP and elastase expression and decrease procollagen synthesis [50, 51]. Macrophages are one of the main sources of MMP, such as MMP-1, MMP-3, MMP-9, and MMP-12 [52, 53]. Baicalin, an active component derived from *Scutellariae Radix*, has been reported to suppress the UVB-induced upregulation of MMP-1 and MMP-3, resulting in inhibition of collagen degradation [22]. In our study, SRT suppressed the infiltration of macrophages compared with the vehicle group at 48 h and 72 h after irradiation (Figure 4(b)). This result suggests that SRT treatment might prevent collagen degradation by inhibiting expression of macrophage-derived MMP. An increase in macrophages was observed from 24 h after irradiation in the vehicle group; however, a reduction of density of collagen fibers was detected at only 72 h after irradiation. It was reported that, in the human skin, acute UV irradiation reduced expression of receptor involved in cellular collagen uptake at 24 and 48 h after irradiation [9]. The UV-induced inhibition of collagen uptake could explain the time lag between upregulation of macrophages and reduction of collagen density.

This study had several limitations. We administered SRT only before UVB irradiation and observed skin condition until 72 h after UVB irradiation when erythema and TEWL were increased. In tissue analysis, however, IL-1*β* and IL-6 gradually increased from 48 h after irradiation in the SRT group, and the level was higher in the SRT group than in the vehicle group at 72 h after irradiation. This could have occurred because all the active compounds contained in SRT may have been metabolized by 72 h after irradiation. It remains unclear whether SRT inhibited or only delayed UVB-induced photodamage. Whether the SRT effect continues to inhibit UVB-induced skin damage for a longer period than examined here, for example, for 1 week, warrants further investigation. Furthermore, the active compounds with protective effects against photodamage were not identified in this study. The detailed time-dependent study of SRT absorption and metabolism is required to reveal more detailed mechanism.

## 5. Conclusions

**Figure 7:**
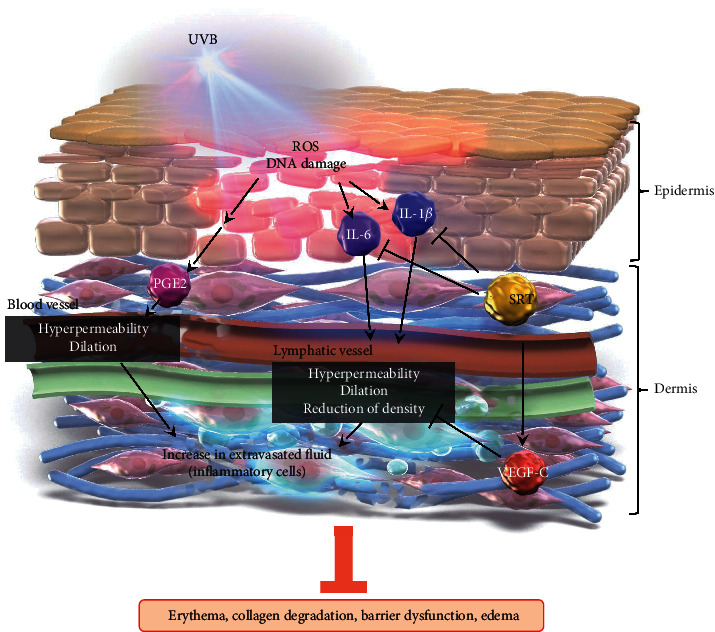
Scheme of SRT inhibition of UVB-induced inflammation and photodamage. SRT: *Saireito* extract; UVB: ultraviolet B; VEGF-C: vascular endothelial growth factor C; PGE2: prostaglandin E2; ROS: reactive oxygen species.

Figure 7 shows a schematic explanation for the mechanism of SRT against UVB-induced photodamage. This study demonstrated that oral pretreatment of SRT suppressed UVB-induced edema formation, cytokine production, and infiltration of inflammatory cells at 24 h after irradiation, suggesting inhibition of early-stage inflammation. Moreover, SRT inhibited the reduction of lymphatic vessel density and upregulated VEGF-C expression. This result suggests that SRT has the potential to improve lymphatic function. SRT also attenuated UVB-induced photodamage represented by increased erythema and TEWL associated with inhibition of ECM degradation at 72 h after irradiation. This result indicates that SRT has a preventive effect against UVB-induced skin damage.

## Figures and Tables

**Table 1:** List of crude drugs composing *Saireito*.

Crude drugs	Plant names	Weight (g)
*Bupleuri Radix*	*Bupleurum falcatum* Linne（*Umbelliferae*）	7.0
*Alismatis Tuber*	*Alisma orientale* Juzepczuk (*Alismataceae*)	6.0
*Pinelliae Tuber*	*Pinellia ternata* Breitenbach (*Araceae*)	5.0
*Poria*	*Wolfiporia cocos* Ryvarden et Gilbertson（*Poria cocos Wolf*） (*Polyporaceae*)	4.5
*Polyporus*	*Polyporus umbellatus* Fries (*Polyporaceae*)	4.5
*Atractylodis Rhizoma*	*Atractylodes japonica* Koidzumi ex Kitamura (*Compositae*)	4.5
*Scutellariae Radix*	*Scutellaria baicalensis* Georgi (*Labiatae*)	3.0
*Ginseng Radix*	*Panax ginseng* C. A. Meyer (*Panax schinseng Nees*)(*Araliaceae*)	3.0
*Ziziphi Fructus*	*Ziziphus jujuba* Miller var. *inermis* Rehder (*Rhamnaceae*)	3.0
*Cinnamomi Cortex*	*Cinnamomum cassia* Blume (*Lauraceae*)	3.0
*Glycyrrhizae Radix*	*Glycyrrhiza uralensis* Fisher or *Glycyrrhiza glabra* Linne (*Leguminosae*)	2.0
*Zingiberis Rhizoma*	*Zingiber officinale* Roscoe (*Zingiberaceae*)	1.0

**Table 2:** Primer sequences used for real-time PCR.

Gene	Primer	Sequence (5⟶3)
IL-1*β*	Forward	TCCAGGATGAGGACATGAGCAC
	Reverse	GAACGTCACACACCAGCAGGTTA

IL-6	Forward	CCACTTCACAAGTCGGAGGCTTA
	Reverse	TGCAAGTGCATCATCGTTGTTC

*β*-Actin	Forward	ACCTTCTACAATGAGCTGCG
	Reverse	CTGGATGGCTACGTACATGG

## Data Availability

The data used to support the findings of this study are available from the corresponding author upon request.
